# Assessing Wearable mHealth Adherence in Underserved Adolescents and its Associations With Physical Activity, Sports, and Safety Perceptions: Prospective Cohort Study

**DOI:** 10.2196/80465

**Published:** 2026-01-29

**Authors:** Annabel Nunez-Gaunaurd, Michele Raya

**Affiliations:** 1 Department of Physical Therapy, Nicole Wertheim College of Nursing and Health Sciences, Florida International University, 11200 SW 8 St, AHC3-424, Miami, FL, 33199, United States, 13053482265; 2 Department of Physical Therapy, Miller School of Medicine, University of Miami, Coral Gables, FL, United States

**Keywords:** mHealth, physical activity, health promotion, obesity, wearable adherence, functional measures, mobile health, wearables, adolescents, school-based

## Abstract

**Background:**

Adolescents from underserved communities, particularly Black and Hispanic youth, engage in lower levels of physical activity (PA), increasing their risk for chronic disease. Conventional interventions often face barriers such as limited access to safe environments. Wearable mobile health technologies offer scalable and context-sensitive solutions; however, predictors of sustained adherence in school-based settings among high-risk populations remain underexplored.

**Objective:**

This study aims to examine the behavioral and contextual predictors of adherence to a consumer-grade wearable PA tracker among underserved high school students.

**Methods:**

In this school-based observational study, 63 students (mean age 14.8, SD 1.17 years) enrolled in physical education received Fitbit devices. Adherence was defined as ≥21 valid days of step count data. Measures included self-reported PA behaviors, neighborhood perceptions, physical fitness (including anthropometrics), and device adherence. Group comparisons were conducted using *t* tests and chi-square tests. Logistic regression was used to identify predictors of adherence.

**Results:**

Overall, 73% (46/63) of participants met the adherence threshold. Adherent students reported fewer days of moderate-to-vigorous PA (2 vs 4 days/week; *P*=.004), lower team sports participation (21/46, 46% vs 12/17, 71%; *P*=.004), and higher perceived neighborhood safety (*P*=.02). In adjusted models, lower PA frequency, greater perceived safety, and neighborhood walkability significantly predicted adherence (*χ*² _6_=16.23*; P*=.01, Nagelkerke *R*²=0.61).

**Conclusions:**

Wearable mobile health technologies show promise for engaging underserved adolescents in PA, particularly those with lower baseline activity and limited access to structured sports. Key predictors of adherence included perceived neighborhood walkability, team sports participation, and prior PA behavior. School-based deployment of wearable devices should emphasize personalized goals and autonomy-supportive strategies to foster sustained engagement and promote PA among high-risk youth.

## Introduction

With national and international guidelines recommending at least 60 minutes of moderate-to-vigorous physical activity (MVPA) daily for children and adolescents, physical inactivity remains a pressing public health concern [1,2]. Regular physical activity (PA) is associated with numerous health benefits, including lower resting heart rate (HR), a key indicator of cardiovascular health [3,4], improved functional fitness [5], and enhanced long-term physical and mental well-being [6]. Despite these benefits, only 22% of adolescents meet the recommended PA guidelines [7], with participation rates significantly lower among Black, Hispanic, and immigrant youth [8]. Many children and adolescents in racially diverse and low-resource neighborhoods lack access to consistent and supportive PA opportunities outside school [9]. Furthermore, the growing financial and time demands placed on parents for youth sports participation [10] have made consistent PA opportunities increasingly inaccessible for many adolescents, particularly those from underserved communities.

Consumer-grade wearable mobile health (mHealth) technologies may offer a scalable, user-friendly means to assess and promote PA engagement [11]. Awareness and use of step-based metrics from wearables can provide an opportunity for self-monitoring, goal setting, and motivation to increase PA behavior and improve health and fitness. For example, typically step-based metrics ranging from 7000 to 11,700 steps per day are associated with meeting Centers for Disease Control and Prevention (CDC)-recommended PA levels [12-14] and with reducing cardiovascular and mortality risks [15]. Conversely, taking fewer than 7000 steps per day may indicate insufficient PA behavior [14]. Wearables can help adolescents become more aware of their PA levels and facilitate tracking of these metrics [16]. There is growing evidence supporting the role of mHealth technologies, including smartphones, apps, and wearables, in promoting positive health behaviors and some improvement in weight status among youth [17]. These devices incorporate behavioral change techniques, such as goal setting, self-monitoring, and feedback [18], and may be particularly useful in school-based programs where environmental constraints can be addressed.

Traditional PA opportunities often rely on structured sports or clinic programs outside of school, which may be inaccessible due to socioeconomic and parental involvement time constraints, limited transportation, and neighborhood safety concerns for underserved youth [19]. Additionally, limited access to safe recreational environments and health-promoting infrastructure further compounds these risks, reinforcing poor PA engagement and long-term health outcomes [20,21]. These limited access to PA opportunities disproportionately affect adolescents from underserved communities, who are at increased risk for deficiencies in motor proficiency, strength, cardiovascular endurance, and overall PA levels, factors closely linked to obesity and poor fitness [22]. Over time, these disparities contribute to the early onset of chronic health conditions such as cardiovascular disease and type 2 diabetes [23]. In contrast, wearable mHealth technologies offer a scalable, low-cost alternative that can be integrated into adolescents’ daily routines, making them particularly promising for populations with limited access to conventional resources.

School-based physical education (PE) programs are a critical platform to provide accessible PA interventions [24]. Wearables integrated into school-based programs can promote autonomy and engagement even in constrained environments. Importantly, low levels of teacher burden have previously been associated with favorable perceptions of feasibility, further supporting the scalability of wearable devices (WD) applicability in school settings [25]. Prior studies suggest that wearables may positively influence PA levels by enhancing self-efficacy and behavioral regulation among adults [26]. Additionally, accuracy and behavioral impact of WD in general have mostly been examined in the adult population [26,27]. Despite the growing popularity of consumer wearables, little is known about long-term adherence to wearable technologies among high school age adolescents from underserved racial and ethnic minority groups [11,28]. There is a significant gap in understanding adherence behavioral patterns among adolescents facing environmental barriers, such as unsafe neighborhoods, low PA engagement, and limited access to recreational spaces.

Recent shifts in PA research emphasize the importance of broader contextual factors, both environmental (ie, social environments, physical settings, and access to resources) and personal (ie, enjoyment, autonomy, and perceived competence), in shaping PA behavior [29]. For example, previous studies found that wearables may be less accessible or sustainable for adults in underserved settings owing to socioeconomic barriers, limited internet access, and concerns about privacy and crime safety [30]. Additionally, research on long-term adherence remains limited, particularly among marginalized youth [31,32]. These contextual challenges may influence how adolescents engage with digital health tools, and the findings from broader populations may not be generalizable to these groups.

Bandura’s Social Cognitive Theory (SCT) provides a behavioral foundation for understanding adherence to WD use, emphasizing how personal factors (self-efficacy and motivation), behavioral patterns (consistent device use), and environmental influences (social support and accessibility) interact to influence health behaviors [33]. The International Classification of Functioning, Disability, and Health (adherence) complements this perspective by situating these behaviors within a biopsychosocial framework, capturing how an individual’s body functions and structures, activities and participation, and contextual factors (personal and environmental) collectively shape overall functioning and engagement in daily life [34]. Together, SCT and the International Classification of Functioning and Disability (ICF) informed the selection of study variables, including self-efficacy, readiness for change, perceived neighborhood safety, and physical fitness and function, allowing for a nuanced exploration of WD adherence and PA engagement among underserved adolescents.

To address the gap in understanding the influence of contextual factors on WD adherence, this study investigated associations among contextual factors, including sociodemographic characteristics, environmental conditions, PA behavior, and adherence to WD, among a racially and socioeconomically diverse cohort of high school students enrolled in a school-based PE program. Guided by the ICF framework, we included predictors, such as self-efficacy, readiness for change, perceived neighborhood safety, and physical fitness, each representing key domains within the ICF [34]. These factors have been validated in prior studies and are particularly relevant to understanding adolescent engagement in PA as they reflect the complex interplay between individual capabilities, motivation, and contextual influences [35]. Specifically, we examine the following: 1. whether demographic, personal, and environmental factors influenced WD adherence; and 2. Whether WD adherence was associated with differences in self-reported PA, fitness, and functional performance outcomes. Our findings aim to inform the design of future school-based wearable interventions that effectively engage adolescents from historically marginalized populations and promote PA through sustained behavioral changes.

## Methods

### Overview

This prospective cohort study was conducted in collaboration with local nonprofit organizations and public-school administrators. Participants were recruited using a purposive sampling strategy designed to ensure the representation of racially and socioeconomically diverse adolescents in an underserved community. School-based physical therapists and PE teachers facilitated recruitment through in-class announcements and take-home informational flyers distributed during sessions of state-mandated Health Opportunities Through Physical Education (HOPE) courses. This course emphasizes lifelong fitness, healthy behavior, and personal responsibility [36]. All students enrolled in the HOPE were invited to participate, minimizing selection bias by including students regardless of their prior PA engagement or academic standing. To reduce sampling bias and ensure equitable participation, parent consent and student assent materials emphasized the voluntary nature of the study, clarified that participation would not affect academic standing, and explained the privacy protections in place. Materials were designed to be culturally appropriate and accessible, with bilingual staff available to address questions for parents or guardians. These steps were taken to minimize the likelihood that guardians would restrict participation based on personal beliefs, misconceptions about wearable technology, or concerns about data privacy.

Although the school served as the central hub for recruitment and data collection, participants wore WD during and outside school hours, enabling continuous monitoring of PA across multiple contexts, including home and community settings. Predictors of adherence, such as neighborhood safety, team sports participation, and self-reported PA behaviors, reflect influences beyond the school setting. Nevertheless, the school played a pivotal role in supporting this study through structured integration of the PE curriculum, device distribution, and ongoing student engagement.

### Study Timeline

Data collection spanned the entire academic year, with baseline assessments conducted in the first quarter and follow-up assessments conducted in the final quarter. This extended duration enabled evaluation of adherence to WD use throughout the academic year.

### Procedures

Baseline assessments were conducted over 2 PE class sessions. Day 1 included demographic and PA surveys, and day 2 involved physical performance testing. In collaboration with PE instructors and school-based physical therapists, students received individualized step-count goals, starting with a 10% weekly increase from their baseline average. PE teachers provided weekly reminders to wear and synchronize their Fitbit devices. Measures such as self-efficacy and readiness for change were selected based on their established predictive value in adolescent PA engagement, as supported by Bandura’s SCT [33] and integration into the WHO-ICF framework. Environmental variables were included to reflect contextual influences consistent with ICF’s socio-ecological approach. Table 1 provides a summary of variables assessed and associated theoretical construct.

**Table 1.** Summary of predictors, data sources, and theoretical frameworks.

Variables	Outcome measures	Theoretical framework
Demographics	Survey during PE^a^ class	Personal factors (ICF)^b^
Self-report-physical activity level	PACE+^c^ survey [37,38] Fitbit Flex [39,40]	Activities and participation (ICF) Behavioral capability and self-regulation (SCT)^d^
Physical activity level	Fitbit Flex [39,40]	Activities and participation (ICF) Self-monitoring and reinforcement (SCT)
Wearable device adherence	Fitbit Flex via Fitabase [39,40]	Activities and participation (ICF) Self-regulation reinforcement (SCT)
Self-efficacy and readiness for change	PACE+ Survey [37,38,41].	Personal factors (ICF) Self-efficacy and expectations (SCT)
Social support and enjoyment	PACE+ Survey [42]	Environmental factors (ICF) Social support and reinforcement (SCT)
MVPA frequency-self-report	PACE+ Survey [42]	Activities and participation (ICF) Behavioral capability and self-regulation (SCT)
Team sports participation	Youth Risk Behavior Survey [43-45]	Activities and participation (ICF) Observational learning and social support (SCT)
Neighborhood walkability	NEWS-Y Scale [46]	Environmental factors (ICF)
Anthropometrics	Height, weight, and waist circumference [47,48]	Body functions and structures (ICF)
Vital signs	Heart rate and blood pressure [49]	Body functions and structures (ICF)
Cardiovascular endurance	6-minute walk test [50,51].	Body Functions and Activities (ICF)
Functional mobility	Timed up and down stairs	Activities and participation (ICF)
Balance	Single-leg stance test [52]	Body functions and activities (ICF)
Upper-body strength	90° push-ups [53].	Body functions and activities (ICF)

a PE: physical education.

b ICF: International Classification of Functioning and Disability.

c PACE: Pollution Abatement Costs and Expenditures.

d SCT: Social Cognitive Theory.

### WD Adherence

Participants received a Fitbit Flex activity tracker and information on device use, care, and data synchronization from school-based physical therapists. These devices contain accelerometers and have demonstrated moderate to excellent validity compared to research-grade accelerometers in youth [39,40]. The Fitbit data were uploaded to Fitabase, a research platform for remote device monitoring. The weekly averages of daily steps were captured using Fitbit Flex devices [54]. Adherence days were defined as days with at least 10 hours of HR monitoring data [55], and Adherence week was defined as a week with at least 3 valid days of WD use [41]. A 21-day period of WD use stratified participants as adherent or nonadherent WD users. This threshold was chosen based on prior studies that defined high adherence to WD use as achieving at least 70% of the recommended daily use over a 30 day period [56] and systematic review benchmarks of 10 hours per day criteria for high adherence to daily use [41]. These timeframes were selected to balance early feasibility with theoretical relevance, particularly in adolescent populations, where motivation and environmental influences may fluctuate.

### PA and Psychosocial Measures

PA behavior and psychosocial constructs were assessed using a patient-centered assessment and counseling for exercise and nutrition (Pollution Abatement Costs and Expenditures) survey. This validated instrument includes items on decisional balance, self-efficacy, enjoyment, and social support [37,38]. It has demonstrated acceptable reliability (ICC=0.60‐0.82) and moderate validity with accelerometer-derived MVPA (*r*=0.27‐0.40) [41]. Additionally, the Pollution Abatement Costs and Expenditures Survey assessed all participants on: days per week of 60 minutes of MVPA, readiness for change, and perceived peer or family support [42-44]. Lastly, team sports participation was measured using the 2013 Youth Risk Behavior Survey, which has shown substantial reliability (κ=0.61‐0.91) [45].

### Neighborhood Walkability

Perceived neighborhood environment was assessed using the Neighborhood Environment Walkability Scale for Youth (NEWS-Y) [46]. The subscales measure safety from crime, traffic hazards, and access to land. Items were rated on a 4-point Likert scale, with higher scores indicating lower walkability. The NEWS-Y has demonstrated good test-retest reliability (ICC=0.56‐0.87) and validity, as evidenced by associations with walking behaviors among youth [46].

### Clinical Assessments

To evaluate physical fitness and functional performance, a series of standardized assessments was administered during PE class sessions by trained physical therapists. These measures were selected based on their reliability, validity, and relevance to the adolescent population.

### Anthropometrics and Vital Signs

Height, weight, and waist circumference data were collected using standardized procedures [47]. BMI was calculated using CDC growth charts. The waist-to-height ratio (WtHR) was calculated; a ratio ≥0.5 indicated elevated central adiposity [48]. Resting HR and blood pressure were measured using standard clinical procedures and equipment [49].

### Functional Fitness Assessments

Cardiovascular endurance was assessed using the 6-minute walk test, a submaximal exercise test that measures the distance an individual can walk in 6 minutes. The 6-minute walk test is widely used in pediatric populations and has demonstrated excellent reliability (ICC=0.94) and strong validity for estimating aerobic capacity [50,51].

Functional mobility was assessed using the timed up and go test, which evaluates lower-extremity strength, coordination, and mobility. Participants were timed as they ascended and descended stairs. The timed up and down stairs test has shown excellent reliability (ICC=0.978‐0.999) and correlates well with other mobility and balance measures [57].

Balance was assessed using the timed single-leg stance test, in which participants stood on one leg for as long as possible without support. This test provides insight into postural control and neuromuscular stability and demonstrates acceptable reliability in youth populations [52].

Upper-body strength and endurance were assessed using 90° push-ups, in which participants performed push-ups until exhaustion with proper form. This test is a reliable indicator of muscular endurance (ICC=0.93) and has demonstrated moderate validity when compared to bench press performance (*r*=0.64; males and *r*=0.28; females) [53].

### Data Analysis

Statistical analyses were performed using SPSS v28. Descriptive statistics were used to characterize the participants. Chi-square and independent *t* tests were used to compare the adherence groups in terms of demographic and outcome variables. Pearson correlation coefficient was used to assess the association between PA behaviors and psychosocial variables. Relationships were classified by effect size: 0.00‐0.25 (none), 0.25‐0.50 (weak), 0.50‐0.75 (moderate), and >0.75 (strong) [58]. Binomial logistic regression was used to examine the associations between WD adherence and demographic, behavioral, and environmental predictors, including PA level, obesity status, perceived neighborhood safety, and nativity. A priori power analysis using G*Power (v3.1.9.6) determined a minimum sample size of 57 to detect medium effect sizes (Cohen *d*=0.5, f^2^=0.30) with *α*=.05 and 80% power.

### Ethical Considerations

This study was approved by the Western Institutional Review Board and the Research Ethics Committee of the County School Board. Informed consent was obtained from all the participants and their guardians. The eligibility criteria were high school adolescents aged 14‐18 years who were enrolled in a HOPE course. Baseline assessments were conducted in the first academic quarter, and follow-up assessments were conducted in the final quarter. Surveys and physical fitness tests were conducted during the PE class. Race, ethnicity, and nativity (US or foreign-born) were self-reported. All collected data were stored on secure, password‑protected servers; identifiable information was removed prior to analysis to ensure participant privacy and confidentiality. No financial or academic compensation was provided, and participation had no effect on course grades.

## Results

### Cohort Characteristics

Participant characteristics by WD adherence (N=63) are presented in Table 2. Of the total sample, 73% (46/63) met the WD adherence criteria. The average age was 14.8 (SD 1.17) years, with the majority in ninth grade (51/63, 81%). More than half of the participants were female (35/63, 56%), and 73% (46/63) received free or reduced lunch. Most were Black or African Americans (40/63, 72%), followed by Hispanics (16/63, 42%). Sixteen percent (10/63) were foreign-born, and 37% (23/63) reported maternal nativity outside the United States. The average BMI and WtHR for the sample were 23.06 (SD 4.62) kg/m² and 0.45 (SD 0.07), respectively.

**Table 2.** Demographic characteristics by wearable adherence, using wearable device (WD) and not using wearable device (N-WD).

Demographic characteristics	Total (N=63)	WD (n=46)	N-WD (n=17)	*P* value^a^
Age (years), mean (SD)	14.79 (1.17)	14.67 (1.06)	15.11 (1.41)	.13
Sex, n (%)				.41
Male	28 (44.4)	19 (41)	9 (53)	
Female	35 (55.6)	27 (59)	8 (47)	
Grade level, n (%)				.20
9th	51 (81)	40 (87)	11 (65)	
10th	2 (3.2)	1 (2.2)	1 (5.9)	
11th	3 (4.8)	1 (2.2)	2 (11.11)	
12th	7 (11.1)	4 (8.9)	3 (16.7)	
Socioeconomics, n (%)				
Free or reduced lunch	46 (73)	—^b^	—	—
Race and ethnicity, n (%)				
Black or African American	40 (73)	28 (72)	12 (75)	.55
Hispanic	16 (42)	14 (47)	2 (25)	.25
Student birth country (non-US)	10 (16)	9 (21)	1 (7)	.24
Parents birth country (1‐2, non-US)	26 (41)	22 (48)	4 (24)	.07
Maternal birth country, n (%)				—
United States	30 (48)	19 (41)	11 (65)	
Bahamas	1 (1.6)	1 (2.2)	—	
Brazil	3 (4.8)	3 (6.5)	—	
Dominican Republic	1 (1.6)	1 (2)	—	
Ecuador	1 (1.6)	0	1 (7)	
Germany	1 (1.6)	1 (2)	—	
Guatemala	1 (1.6)	1 (2)	—	
Haiti	8 (13)	7 (15)	1 (7)	
Honduras	1 (1.6)	1 (2)	—	
Jamaica	2 (3)	2 (4)	—	
Mexico	3 (4.8)	2 (4)	1 (7)	
Peru	1 (1.6)	1 (2)	—	
Country not reported	10 (16)	7 (15)	3 (18)	

a Chi-square *P* value of ≤.05 considered statistically significant

b Not applicable.

### Anthropometric Characteristics

As detailed in Table 3, 19% of participants were classified as overweight (12/63) and 15.9% were classified as obese (10/63). Also, 21% (13/63) had a WtHR >0.5, indicating elevated cardiometabolic risk. Significant differences between the WD adherence groups were found in resting HR (76.4 vs 67.4 bpm; *P*=.009) and single-leg stance duration (57 vs 59 s; *P*=.03). No statistically significant differences were found in BMI, WtHR, blood pressure, 6 MWT, timed up and down stairs, push-ups, or sit-to-stand performance.

**Table 3.** Health outcomes characteristics by wearable adherence status, using wearable device (WD) and not using wearable device (N-WD).

Characteristics	Total (N=63)	WD (n=46)	N-WD (n=17)	*P* value^a^
Anthropometrics				
BMI (kg/m^2^), mean (SD)	23.06 (4.62)	23.16 (4.9)	22.79 (3.9)	.78
BMI-for-age (percentile) , mean (SD)	66.25 (9.02)	66.1 (30.7)	66.64 (24.6)	.96
Underweight (<5 percentile), n (%)	3 (4.8)	3 (6.5)	0 (0)	.38
Healthy weight (5-<85 percentile), n (%)	36 (57)	25 (54)	11 (65)	.38
Overweight (≧85 percentile but ≦95 percentile), n (%)	12 (19)	8 (17)	4 (24)	.38
Obese (≧95 percentile*),* n (%)	10 (15.9)	9 (20)	1 (6)	.38
WHtR^b^, mean (SD)	0.45 (0.07)	0.46 (0.08)	0.44 (0.04)	.20
≦0.5 normal risk, n (%)	49 (77.8)	34 (74)	15 (88)	.09
>0.5 higher risk, n (%)	13 (21)	12 (26)	1 (6)	.09
Vitals, mean (SD)				
Resting HR^c^ (beats/min)	73.95 (14.3)	76.4 (15.3)	67.4 (9.1)	.01
SBP^d^ (mm Hg)	114.7 (14.4)	115.2 (14.7)	113.4 (14.1)	.68
DBP^e^ (mm Hg)	73.34 (12.9)	74.2 (13.6)	71.1 (10.9)	.42
Performance health outcomes, mean (SD)				
6MWT^f^ (m)	503.14 (62.72)	496.5 (66.1)	520.6 (50.6)	.19
SLS^g^ (right+left, 60s max)	57.41 (6.21)	56.9 (6.9)	59.4 (1.4)	.03
Timed up and down stairs (s)	9.27 (3.68)	9.6 (4.1)	8.3 (1.5)	.23
Push-ups repetitions	13.77 (10.36)	12.6 (10.5)	18 (8.8)	.11
Sit-to-stand repetitions per minute	36.7 (7.36)	36.13 (7.6)	38.47 (6.52)	.29

a *P* values (*P*<.05) were calculated on the basis of *t* test, *P* value of ≤.05 considered statistically significant.

b WHtR: waist to height ratio.

c HR: heart rate.

d SBP: systolic blood pressure.

e DBP: diastolic blood pressure.

f 6MWT: six-minute walk test.

g SLS: single-leg stance.

### PA and Environmental Perceptions

Table 4 summarizes the psychosocial and environmental characteristics based on WD adherence. On average, participants reported engaging in ≥60 minutes of MVPA at 2.86 (SD 2.17) days per week. Only 13% (8/63) participants met the daily MVPA guidelines, with males being more likely than females to do so (7/28, 25% vs 2/35, 5.7%; *P*=.06). Notably, 24% (15/63) of the participants reported no MVPA during the week, with inactivity being more prevalent among female adolescents (13/35, 36% vs 3/28, 12%). Participation in team sports was positively associated with MVPA; among the 33 adolescents who participated in sports, only 3 reported no MVPA. However, WD-adherent participants reported fewer days of MVPA (2 d vs 4 d; *P*=.004), fewer days of muscle-strengthening activities (3 vs 4.8 d; *P*=.004), and lower team sports participation (21/46, 46% vs 12/17, 71%; *P*=.004) compared to their nonadherent peers. No significant differences were found in psychosocial measures such as self-efficacy, family or friend support, or decisional balance. Interestingly, adherent participants perceived their neighborhoods as safer, with lower scores for crime (1.7 vs 2.4; *P*= .02), traffic concerns (2.1 vs 2.4; *P*=.04), and reported greater access to land-use features (3.1 vs 2.9; *P*=.04).

**Table 4.** Physical activity–related psychosocial characteristics and perceptions of neighborhood crime and walkability by wearable adherence status, using wearable device and not using wearable device.

Characteristics	Total (N=63)	WD^a^ (n=46)	N-WD^b^ (n=17)	*P^c^* value
PA^d^ steps per day				
PA wearables, average steps/day, mean (SD)	—^e^	7875 (2696.23)	—	—
PA<10,000 steps/day, n (%)	—	38 (83)	—	—
PA <7000 steps/day, n (%)	—	19 (41)	—	—
PA <5000 steps/day, n (%)	—	7 (15)	—	—
PA self-report measures				
PA 60 min-MVPA^f^ days*<7* days, n (%)^g^	54 (86)	42 (91)	12 (71)	.04
PA 60 min-MVPA days*<3* days, n (%)^g^	29 (46)	26 (57)	3 (18)	.006
PA 60 min-MVPA days 0 days, n (%)	15 (24)	13 (28)	2 (12)	.17
PA 60 min-MVPA days, mean (SD)	2.86 (2.17)	2.39 (2.07)	4.12 (1.97)	.004
PA muscle strengthening days, mean (SD)	3.49 (2.29)	3 (2.02)	4.82 (2.48)	.004
PA team sports participation, n (%)^g^	33 (52)	21 (46)	12 (71)	.08
PA team sports (number of teams), mean (SD)	1.94 (1.11)	1.76 (1.02)	2.41 (1.22)	.02
PA stage of change, mean (SD)	2.89 (1.01)	2.78 (.97)	3.23 (1.10)	.06
PA con, mean (SD)	1.81 (.75)	1.82 (.77)	1.76 (.71)	.40
PA pro, mean (SD)	3.64 (1.01)	3.77 (1)	3.29 (.97)	.06
PA self-efficacy, mean (SD)	3.12 (1.06)	3.04 (1.12)	3.37 (.82)	.15
PA-family support, mean (SD)	2.68 (1.34)	2.57 (1.12)	3 (1.42)	.12
PA-friend support, mean (SD)	2.95 (1.05)	2.90 (0.91)	3.09 (0.86)	.48
Neighborhood Walkability Scale, mean (SD)				
Land-use mix–diversity^h^	3.77 (0.83)	3.80 (0.79)	3.69 (0.96)	.65
Neighborhood recreation facilities^h^	3.61 (0.95)	3.59 (0.94)	3.66 (1)	.80
Residential density^h^	3.14 (1.10)	3.18 (1.10)	3.07 (1.14)	.74
Land-use mix–access^h^	3.04 (0.41)	3.10 (0.39)	2.86 (0.40)	.04
Street connectivity^h^	2.95 (0.61)	3 (0.58)	2.81 (0.68)	.30
Walking or cycling facilities^h^	2.99 (0.58)	2.91 (0.58)	3.20 (0.51)	.08
Neighborhood esthetics^h^	2.68 (0.58)	2.73 (0.60)	2.52 (0.51)	.22
Pedestrian and automobile traffic safety^i^	2.23 (0.41)	2.17 (0.43)	2.41 (0.31)	.04
Crime safety^i^	1.79 (0.68)	1.68 (0.61)	2.13 (0.78)	.02

a WD: wearable device.

b N-WD: not-using wearable device.

c *P* value of ≤.05 considered statistically significant.

d PA: physical activity.

e Not available.

f Activities require moderate physical effort and cause small increases in breathing or heart rate to vigorous physical activities that require hard physical effort and cause large increases in breathing or heart rate.

g Pre-not meeting Center for Disease Control and Prevention recommendations.

h Higher score=higher walkability.

i Higher score=lower walkability.

### Predictors of Wearable Adherence

The results of the binomial logistic regression analysis in Table 5 indicate that the model was significant (*χ*² _6_=16.23; *P*=.01), explaining 61% of the variance (Nagelkerke *R*²). Overall, 2 significant predictors emerged: perceived neighborhood crime safety and MVPA frequency. Adolescents engaging in <3 days per week of MVPA had significantly higher odds of WD adherence (OR 6.1, 95% CI 1.5-24) than those who met or exceeded the 3-day threshold. These findings suggest that lower levels of PA and perceptions of neighborhood safety may influence engagement with WD.

**Table 5.** Logistic regression predicting the likelihood of wearable device adherence.

Variables		β	SE	Wald (*df*)	Sig.	Exp (β)	95% CI for Exp (β)
Step 1							
Sex		−2.685	2.238	1.439 (1)	0.230	0.068	0.001-5.486
Ethnicity		1.478	1.498	0.974 (1)	0.324	4.386	0.233-82.609
Obesity status		3.036	2.178	1.943 (1)	0.163	20.813	0.291-1486.464
HR^a^ (rest)		.057	0.069	0.685 (1)	0.408	1.059	0.925-1.211
PA^b^ self-report		−1.234	0.620	3.957 (1)	0.047	0.291	0.086-0.982
Crime safety		−2.442	1.094	4.986 (1)	0.026	0.087	0.010-0.742
Constant		3.966	6.569	0.365	0.546	52.798	—^c^

a HR: heart rate.

b PA: physical activity.

c Not applicable.

Despite the early engagement, adherence to Fitbit monitoring declined sharply over time. Of the 63 students who initiated device use, 42 (67%) maintained adherence for at least 2 months. By month 3, retention dropped to 29 students (46%), and by month 4, only 21 students (33%) remained. Long-term adherence was minimal: 9 students (14%) persisted through month 5 and only 3 (5%) continued through month 7. This trajectory reflects a steep early decline followed by a gradual taper, underscoring the challenge of sustaining engagement beyond the initial months.

## Discussion

### Principal Findings

This study offers novel insights into WD adherence among underserved adolescents in a school-based setting, thus addressing a critical gap in the literature. While prior research has emphasized the role of personal and environmental factors in shaping PA behaviors, primarily focused on homogenous or higher-resource populations [27], few studies have explored how these influences interact with technology engagement in diverse, resource-limited populations. Notably, our findings revealed that adolescents with lower baseline PA, defined as engaging in MVPA fewer than 3 days per week and those least involved in team sports were more likely to adhere to WD use, precisely the group most in need of sustained support. Furthermore, 90% (9/10) of participants with obesity adhered to WD use, underscoring the potential of wearables to engage adolescents at elevated risk for chronic health conditions. Although counterintuitive, this pattern may reflect an opportunity to drive behavior change, wherein previously inactive youth leveraged technology as a catalyst to initiate and sustain PA. These findings align with prior research, such as the RAW-PA study [25], which demonstrated the high acceptability of wearable interventions among adolescents attending socioeconomically disadvantaged schools. To further explore the impact of WD adherence on PA behavior, we conducted a post hoc analysis of step-count trends over the academic year. WD-adherent participants with lower baseline PA demonstrated modest but consistent increases in average daily steps, suggesting a potential behavioral change. Although further research is recommended, these results support the potential for wearables to facilitate engagement and promote sustained improvements in PA levels among adolescents, who are most in need of interventions.

Furthermore, adherence to WD use is often undefined or underreported in adolescent cohorts [59], particularly in resource-limited community-based settings. Our study addressed this gap by defining adherence and examining longitudinal patterns in a population disproportionately affected by obesity and related chronic conditions, thereby distinguishing it from short-term pilot studies. WD adherence was defined as having WD HR data for more than 10 hours per day for at least 3 days of the week for a minimum of 3 weeks, which aligns with benchmarks established in the wearable adherence literature [55,56] and indicates meaningful engagement. Furthermore, the data collection spanned the academic year, providing a flexible timeline for teachers and students to initiate WD use as needed. Although the initial WD engagement was high (46/63, 73%) following deployment, adherence declined sharply after the first month, with only 14% persisting until month 5 and 5% through month 7. Our findings in this underserved population echo more recent systematic reviews reporting mixed results for long-term adherence and call for interventions that combine technology with behavioral support and environmental modifications [60,61]. These results underscore the challenge and opportunity of maintaining engagement beyond the initial novelty period and highlight the need for tailored strategies, such as non–peer-competitive gamification and personalized goal setting.

Our study also revealed a stronger than anticipated association between perceived neighborhood safety and WD adherence. Our findings support the role of baseline PA behavior and environmental perceptions in PA adherence in previous studies, while extending this knowledge to WD use among predominantly Black or African American and Hispanic high school adolescents. Moreover, schools can leverage wearable data to personalize interventions, monitor progress, and foster peer support, thereby enhancing engagement and safety. Integrating wearables into school-based programs that address environmental constraints may offer a promising strategy to promote equitable PA engagement and reduce chronic disease risk [62,63]. Collaborations between schools, policymakers, device developers, and health care professionals are essential to ensure equitable access to supportive technologies and environments [22,64,65]. Beyond behavioral approaches, environmental modifications, such as improving walkability, access to sidewalks, and expanding green spaces, can support sustained PA.

Future school-based studies should explore adaptive strategies with WD within PE and wellness classes. For example, dynamic goal adjustment and real-time feedback help maintain motivation throughout the academic year, ensuring consistent exposure and accountability [66]. Wearables may also complement these efforts by mitigating environmental barriers, enabling self-monitoring, goal setting, and engagement in safe, controlled environments such as homes or schools. To enhance long-term engagement, future interventions should prioritize personalized goal setting over gamified peer competition. Evidence suggests that normative comparisons, such as an arbitrary selection of 10,000 steps/day or competition with peers, may yield only short-term motivation and can negatively impact autonomy [67]. Supporting adolescents in setting individualized goals and encouraging self-referenced progress may foster more sustainable PA habits and better align with their personal values and contexts [67,68]. For adolescents not engaged in team sports, additional motivational strategies, including mentorship and small-group activities, may foster social connections and reduce perceived isolation [69].

Although inconclusive, our study also found higher WD adherence rates among female participants than among their male peers, further highlighting the potential influence of safety-related factors on engagement with wearable technologies and underscoring the need for future research to explore sex-specific motivators and barriers to adherence. Furthermore, our study provided insights into the unique needs of adolescents who are first- or second-generation immigrants navigating acculturation, a group that has been shown to experience sharp declines in PA over time in the United States [70]. Despite their medically underserved status, race, ethnicity, and foreign birth did not significantly influence WD adherence in this study. These findings suggest that WD technology can be a culturally neutral and accessible tool for promoting PA across diverse populations. Further research should disaggregate data by generational status and language proficiency to better support first-generation and non–English-speaking youths [71].

### Limitations

This study offers valuable insights into WD adherence among underserved adolescents; however, it had several limitations that should be addressed. The unexpectedly strong influence of perceived neighborhood safety may reflect regional or demographic differences, including potential sampling bias, and suggests that environmental safety perceptions play a more prominent role in technology engagement than previously recognized. The reliance on self-report measures introduces potential recall and social desirability biases, and the cross-sectional design limits causal inferences. The relatively small sample size and concentration of participants within a single school year (51/63, 81% in ninth grade) further constrain generalizability. This age clustering may obscure developmental differences in PA behavior and technology engagement across adolescence. Additionally, variability in school schedules, seasonal factors, and academic demands may influence adherence patterns and should be considered in future studies. Future studies should aim for broader age representations and larger multisite samples to enhance external validity and capture developmental variability.

This study highlights the potential of wearable mHealth technologies to engage underserved adolescents in PA, particularly those with lower baseline activity levels and limited access to structured sports. Our findings suggest that WD adherence is influenced not only by individual behaviors but also by contextual factors, such as perceived neighborhood safety, an often-overlooked determinant of technology engagement among youth. The counterintuitive finding that adolescents with lower baseline PA levels were more likely to adhere to WD use warrants further investigation. Future research should explore mHealth access, usability barriers, and motivational factors for sustained WD use to improve PA. Anecdotal reports from teachers indicated that some students faced challenges with WD use, including limited Wi-Fi connectivity at home or school, forgetfulness, and declining interest over time. These barriers merit further exploration to inform the design of more accessible and engaging mHealth interventions. Despite being at a critical developmental stage for establishing lifelong health behaviors, older adolescents (aged 14 y‐19 y) remain underrepresented in mHealth research. Multischool collaborations and standardized adherence metrics are essential for scaling wearable-based interventions and strengthening cross-study comparisons.

### Conclusions

This study highlights the potential of wearable mHealth technologies to engage underserved adolescents in PA, particularly those with lower baseline activity levels and limited access to structured sports. Adherence to WD use was significantly associated with contextual factors, including perceived neighborhood walkability, team sports participation, and prior PA behavior. These findings underscore the importance of designing school-based WD deployment that prioritizes autonomy-supportive strategies, such as personalized goal-setting and self-referenced progress to initiate and sustain PA opportunities. Moving beyond normative comparisons and peer-based competition may enhance sustained engagement and promote equitable health outcomes among high-risk youth.

## Abbreviations

HOPE: Health Opportunities through Physical Education
HR: heart rate
ICF: International Classification of Functioning and Disability
mHealth: mobile health
MVPA: moderate-to-vigorous physical activity
PA: physical activity
PE: physical education
SCT: Social Cognitive Theory
WD: wearable device
WtHR: waist-to-height ratio
